# Postural deficiencies prevalence and correlation with foot conditions, body composition, and coordination, in Romanian preadolescents children: descriptive observational study

**DOI:** 10.3389/fped.2025.1621792

**Published:** 2025-10-14

**Authors:** Silviu Vlad, Doriana I. Ciobanu, Julia Fulop, Nicola Matei, Dana I. Cristea, Mariana Szabo-Alexi, Florin N. Blaga, Dorina Ianc, Alexandru B. Ilies

**Affiliations:** ^1^ Department of Surgical Specialties, Faculty of Medicine and Pharmacy, University of Oradea, Oradea, Romania; ^2^ Department of Physical Education, Sport and Physiotherapy, Human Performance Research Center, University of Oradea, Oradea, Romania; ^3^ Help&Care Association, Oradea, Romania; ^4^ Orthopedics and Traumatology Department, Avram Iancu Military Hospital, Oradea, Romania; ^5^ Doctoral School of Biomedical Sciences, Faculty of Medicine and Pharmacy, University of Oradea, Oradea, Romania

**Keywords:** posture, spine, valgus ankle, baropodometry, preadolescents

## Abstract

**Background:**

Correct posture during preadolescence is crucial for harmonious physical development and long-term musculoskeletal health. The examination of spinal and lower limb deficiencies in this age group represents a highly relevant and underexplored topic.

**Objective:**

To determine the prevalence of postural deficiencies among Romanian preadolescents and to assess their correlations with body composition, coordination, and foot morphology.

**Methods:**

A total of 983 children aged 8–12 years (507 boys, 51.6%; 476 girls, 48.4%) were recruited from six middle schools in Oradea, Bihor County, Romania. Postural assessment followed Kendall et al.'s protocol using a plumb line and grid chart. Plantar pressure and center of gravity displacement were evaluated through baropodometry, while general coordination was assessed using the Matorin test.

**Results:**

Forward head posture was the most prevalent deficiency (641/983, 65.2%). Boys exhibited a higher prevalence of kyphosis (*n* = 448, 52.3%) compared with girls (*n* = 368,40.4%), while scoliosis occurred more frequent in girls (*n* = 306, 33.6%) vs. (*n* = 257, 26.1%). Significant correlations were observed between ankle valgus and scoliosis [x^2^(1) = 7.87, *p* = .005], flatfoot and scoliosis [x^2^(1) = 7.87, *p* = .005], and flatfoot and coordination deficits [x^2^(3) = 22.96, *p* = .005].

**Conclusions:**

Forward head posture emerged as the most common spinal deficiency. Notable associations were identified between body composition and kyphosis, hyperlordosis, and ankle valgus, as well as between flatfoot, scoliosis, and impaired coordination. These findings underscore the importance of early detection and the implementation of targeted prevention programs to address postural deficiencies during childhood.

## Introduction

Correct posture during preadolescence is fundamental for balanced physical development and long-term health. Regular physical activity appropriate ergonomics, and healthy lifestyle habits play essential roles in maintaining postural alignment. Children should be encouraged to monitor their posture and to take frequent breaks during sedentary activities in order to prevent muscle stiffness (1). Proper posture reflects musculoskeletal balance which protects anatomical structures from injury or deformity, whereas poor posture, characterised by improper alignment, increases tension in supporting structures and negatively affects balance. Posture is influenced by multiple factors, including age, gender, somatic structure, psychological state, lifestyle, and level of physical activity (2).

Postural deviations are increasingly recognised as a significant public health concern among school-aged children. Epidemiological studies from Romania reporting a high prevalence of spinal misalignments, including scoliosis, kyphosis, and lordosis, frequently identified through school-based screenings using visual methods such as the plumb line (3, 4). Similar findings have been documented in other European countries, where between one-third and more than half of children and adolescents exhibit some form of postural deficiency, underscoring the widespread nature of the problem (5, 6). Beyond their clinical relevance, these deviations carry important social implications: they may affect physical performance, self-image, and quality of life during a sensitive developmental stage, while also predisposing individuals to musculoskeletal pain and dysfunction in adulthood. Consequently, the early identification and monitoring of postural deviations represent not only a medical but also a social priority, highlighting the need for reliable assessment methods and preventive interventions within the school environment. Throughout life, and particularly during prepubertal and pubertal phases, body posture undergoes significant changes as a result of multiple factors. Environmental influences, sedentary behaviour, reduced physical activity, and inadequate diet exert increasingly negative effects on posture (2). Among these influences, childhood obesity has emerged as a major concern, profoundly affecting postural indices. Contributing elements include reduced physical activity and prolonged screen time, trends that have been further intensified by the recent pandemic. Increased body mass index (BMI) has been associated with decreased stability, alterations of the pelvic axis, exaggerated lumbar lordosis, abdominal prominence, internal hip rotation, valgus knees, and flat feet (7).

Research has demonstrated correlations between body composition and postural or foot abnormalities in both children and adults (8–10). Elevated fat mass is associated with deviations from normal posture, whereas a higher percentage of skeletal muscle mass and fat-free mass correlates with fewer postural anomalies (8). Greater total fat mass and fat percentage are also linked to a more pronated foot posture (9). Jorgić et al. also highlighted the consistent influence of body composition parameters—such as fat mass and skeletal muscle percentage—on posture among children from different environments (10).

The relationship between posture and balance in children has been extensively studied. Ludwig et al. reported weak but significant correlation between age, BMI, and sway path length, but no significant association between posture parameters and sway path length (11). In a subsequent study, Ludwig also examined the influence of age on posture and balance control in a healthy pediatric population, finding subtle age-related changes (12).

Foot deformities and their relationship to balance reactions have also received attention. Wilczyński and Paprocki found significant correlations between foot abnormalities and balance deficits in school-aged children (13). Wilczyński et al. subsequently confirmed the association between postural variables and postural stability, observing that poorer balance is correlated with more pronounced postural defects (14).

Research into the interplay between posture and balance has expanded considerably. Azevedo et al. analysed the relationship between sagittal spinal angles and static equilibrium among 1,154 subjects, concluding that spinal postural angles are poor predictors of stabilometric outcomes (15). Nevertheless, posture remains fundamental for maintaining proprioception and coordinating movement, with posture and balance control systems operating in concert during locomotion and object manipulation (16).

Postural control relies on sensory input from visual, vestibular, and somatosensory systems, which integrate environmental information during motor planning and provide continuous feedback for movement adjustment (17). While previous research focused primarily on balance and posture separately, recent studies are beginning to explore potential links between poor posture, foot deformities, and coordination. For instance, variability in lower limb inter-joint coordination has been observed during gait in children with flexible flat feet (18). Moreover, Azevedo et al. emphasized the critical role of the foot in postural stability, through mechanical support, muscle coactivation, and sensory feedback from plantar mechanoreceptors (15).

Given the importance of early detection, somatoscopic assessment has become a widely used methods for identifying postural deficiencies, particularly spinal deformities (19). The existing literature underscores the complex interrelationship between posture and balance, yet important gaps remain in in understanding the specific correlations between common spinal deficiencies and foot deformities in preadolescents.

Current studies have mainly balance and its association with general posture parameters.

The aim of this study is therefore to estimate the prevalence of spinal and foot postural deficiencies among preadolescents and to determine whether correlations exist between bad posture, body composition and coordination as well as between foot conditions and spinal postural deficiencies.

## Methods

### Study area

Data collection was conducted in Oradea, Bihor County, Romania, an important economic and cultural center located in the northwestern part of Romania, Crisana region, with a population of approximately 245,537 inhabitants.

The district covers an area of 115,6 km2 with an estimated population of 245,537 inhabitants according to the 2021 census, resulting in a population density of approximately 1,585 inhabitants/km, which places Oradea as the 9th most populous city in Romania.

According to the information available for the 2023–2024 school year, Oradea's educational network includes17 middle schools (14 public and 3 private), with an average number of 850 children/units, providing a representative sample for assessing postural health in this age group.

### Study design and period

This descriptive observational study was conducted in a cross-sectional manner, without intervention, between February to June 2024. The objective was to assess the prevalence of spine and feet postural deficiencies among preadolescents. The evaluation was performed by an experienced team of two physical therapists from Help&Care Association, Oradea, Romania.

### Study population

The study population comprised preadolescent children enrolled in the middle school network of Oradea, Bihor County, Romania.

### Sample size

To establish the sample size, the total number of children in all 17 middle schools of Oradea (approximately 14,450, with an average of 850 pupils per school) was used as the reference population. The calculation assumed a 95% confidence level, a 5% margin of error, and a population proportion of 0.5. Using an online sample size calculator (https://www.calculator.net/sample-size-calculator), the minimum recommended sample size was determined to be 357. Ultimately, 983 participants were included in this study, exceeding the minimum requirement.

### Sampling procedures

A simple random sampling method was used for the recruitment process. The study protocol was submitted to the Institutional Review Board (IRB) for ethical approval prior to recruitment. The County School Inspectorate authorised the project, approved its implementation within educational institutions, and sent an invitation to ten local middle schools in Oradea. Enrollment followed a “first come, first served” principle. Six schools agreed to participate; four either declined or did not respond. No schools withdrew after enrollment.

The principals of participating schools distributed invitation to homeroom teachers of classes with pupils aged 8–12 years. The materials described the study's aims, procedures, evaluation process, and potential risks and benefits, and were forwarded to parents or legal guardians. They were asked to review the informations and provide written consent for their child's participation within one week.

On average, five classes per school (*n* = 182 children) agreed to participate. Based on parental consent, homeroom teacher compiled lists of students who wished to participate and submitted them to the project coordinator.

In total, 1,092 potential participants were screened for eligibility, with inclusion criteria requiring age 8–12 years and the absence of disability, congenital locomotor defects, genetic, hormonal, or neuromuscular diseases, and absence of minor musculoskeletal issues. On average, 10% of students per school were excluded owing to dropout or illness.

**Figure 1:**
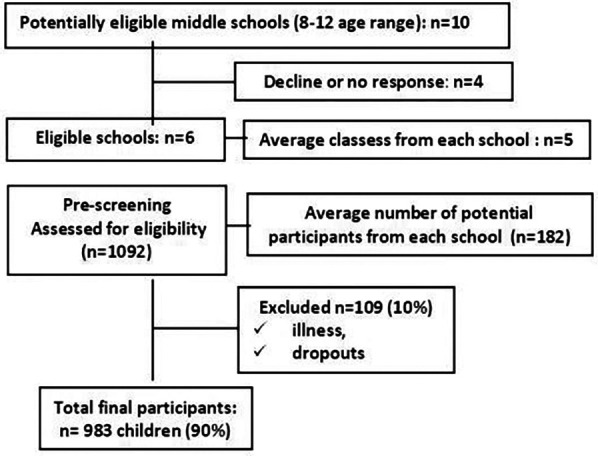
Flowchart of preadolescents recruitment process, in Bihor County, Romania, 2024.

Ultimately, 983 children (average 164 per school; 90% of the initial sample) completed the one-time postural screening session (Figure 1).

### Data analysis and communication of results

Trained professionals analysed the data. Parents received results along with tailored recommendations for exercises or interventions. Certificates of participation were distributed at the end of the study to encourage voluntary involvement and minimize selection bias.

### Data protection and GDPR compliance

Participant identities and data were anonymized and securely stored, with access restricted to the research team. All procedures adhered to the General Data Protection Regulation (GDPR).

### Blinding procedure

To reduce bias and enhance reliability, partial blinding was applied. Participants were not informed of the specific hypotheses to limit response bias. Data analysts worked with de-identified datasets to prevent unconscious bias. These measures strengthened objectivity, validity, and reproducibility.

### Participants

The final cohort comprised 983 children aged 8–12 years, mean 10.03 (SD 1.41). Height ranged from 116 to 179 cm, mean 145.15 (SD 11.76), weight from 18.4 to 118.3 kg, mean 41.36 (SD 13.47) and body mass index (BMI) from 11 to 168, mean 19.41 (SD 6.44). All were recruited from middle schools in Oradea, western Romania.

The inclusion criteria were: age 8–12 years, no certificate of physical or intellectual disability, no diagnosed syndromes or congenital defects of the locomotor system that might impair psychomotor development, no genetic syndromes, hormonal disorders, neuromuscular diseases, no congenital motor system defects. The exclusion criteria were: the presence of syndromes and congenital musculoskeletal system defects, certificate of physical or intellectual disability, disorders likely to cause pathological posture (e.g., genetic syndromes, hormonal disorders, neuromuscular diseases, congenital defects of the locomotor system, slight musculoskeletal problems), age below 8 and above 12, no written consent for testing.

### Ethical considerations

The study was conducted at the Research Center of Human Performance, Oradea University, in collaboration with Help&Care Association. Approval was obtained from the University Ethics Committee (No. 570/25.04). The study adhered to the Declaration of Helsinki to protect participants’ rights and well-being. Participants were fully informed, and minors provided consent via legal representatives. Confidentiality was ensured by assigning unique codes, with all data stored securely on encrypted servers. No compensation was provided.

### Procedure

Body fat was assessed using the Jackson and Pollock method (20). Three skinfold sites were measured with a skinfold caliper: in girls—triceps, abdomen, thigh; in boys—chest, abdomen, thigh. Body fat percentage was calculated using the Jackson and Pollock 3-site equation. The GIMA brand adipocentimeter was used, which allows a correct assessment of the nutritional status and the sectoral distribution of adipose tissue, dimensions: 16.5 cm × 10 cm × 0.5 cm, weight 22 g.

Posture was examined following Kendall et al.'s protocol, employing a plumb line and a grid chart (5 × 5 cm vertical and horizontal lines) to identify imbalances of the head, trunk and lower limbs (21). Posture was assessed in the sagittal and frontal planes. Children were evaluated individually, standing and wearing appropriate clothing. Each child was instructed to assume a natural, relaxed standing position. The presence or absence of postural deviations was recorded as a dichotomous variable (Yes/No), with deviations defined as protracted or retracted shoulders, forward or backward head position, and inward or outward alignment of the knees or ankles. In view of the presence of gibbosity, a scoliometer was employed to measures the asymmetry of the trunk in scoliosis or the rotation angle of the trunk (22). It is a non-invasive device that is placed on the spine while a person is in a “lean forward” position. A GIMA brand scoliometer was used to confirm the presence of scoliosis. For the purposes of this study, posture analysis results were expressed as the total number of cases for each type of postural deviation.

Intra-rater reliability was assessed by a single evaluator re-analysing the same children, while inter-rater reliability was tested by a second evaluator. Agreement was quantified using Cohen's kappa (k).

Plantar analysis was performed using baropodometry (23). Baropodometry involves the assessment of static and dynamic plantar pressure using a foot pressure plate. When the foot encounters the sensors, a pressure map is generated instantaneously. The signal produced by the sensor activation are recorded and processed to create an image in which pressures magnitudes are represented by different shades of color. For this study, a flexible resistive baropodometric platform (Wiva® Smart FLex Plate-Letsense) was used. The system connects via WI-FI through the BIOMECH ® application (compatible with IOS and Android tablets) or via USB cable to a computer, enabling rapid acquisition of both static and the dynamic measurements (24). The analysis documented the presence or absence of plantar arch flattening and yielded the distribution of anterior, posterior, and lateral load. The following variables were recorded: anterior displacement of the center of gravity (COGA), posterior displacement of the center of gravity (COGP), right displacement of the center of gravity (COGR), left displacement of the center of gravity (COGL). Flattening of the plantar arch has been shown to correlate with pressure ratios across plantar regions (25, 26) and with displacement of the center of gravity (27).

General coordination was evaluated using the Matorin test (28). To perform the test, a circle must be drawn on the ground on which the degrees (0°, 45°, 90°, 135°, 180°, 225°, 270°, 315°, 360°) are noted. The subject places himself in the center of the circle and performed rotational jumps around the longitudinal axis, to the right or to the left. The subject is asked to land in the same place and position as when starting. The degrees of rotation achieved were recorded, with three attempts per child; the best performance was considered.

## Statistical analysis

### Quantitative and qualitative variables

Data analysis was performed using SPSS Evaluation version 15.0.0 (IBM, Oradea, Romania). Means and standard deviations were calculated for quantitative variables, while categorical variables were expressed as percentages. Extreme values were excluded to minimise bias. The normality of quantitative data distribution was tested using the Kolmogorov–Smirnov test. For qualitative variables, the Chi-square test for homogeneity was applied to evaluate whether frequency counts differed significantly between groups according to age and gender.

### Statistical methods

Descriptive statistics were used to characterise the parameters of interest: mean ± standard deviation for ordinal data and percentages for categorical data. To determine whether significant gender-related differences existed in spinal postural deficiencies and foot conditions (flatfoot and ankle valgus), the Pearson Chi-square test was applied. Adjusted Standardised Residuals were computed to identify the specific groups contributing to significant differences.

The Phi coefficient was employed to assess correlations between foot conditions and spinal postural misalignment, with 95% confidence intervals (CIs) reported where appropriate. Point-biserial correlation was used to examine associations between BMI or BFI and the presence of spinal or foot deficiencies. As the analysis involved testing correlations between a dichotomous and a continuous variable, the point-biserial correlation was considered more appropriate than Spearman's correlation. A binomial logistic regression was conducted to examine the effects of age, gender, and BMI on the likelihood of presenting with flatfoot, ankle valgus, forward head posture, kyphosis, hyperlordosis, and scoliosis.

Finally, independent-samples t-tests were performed to compare coordination outcomes and center of gravity (COG) displacements between boys and girls.

## Results

Table 1 presents the characteristics of the study population in terms of age, height, weight, body mass index, body fat index and activity level, both for the entire group and stratified by gender. The preadolescents group comprised 507 boys (51.6%) and 476 girls (48.4%). No significant gender-differences were observed in age, height, weight, BMI, or BFI. With respect to activity level, 476 participants (48.4%) reported regular engagement in sport. Of these, 261 boys (54.8%) and 215 girls (42.3%) engaged in regular sport activity.

**Table 1:** Demographic characteristics for total preadolescents group and according to gender in Bihor County, Romania, 2024.

Demographic characteristics	Total group mean (SD) (*N* = 983)	Boys mean (SD) (*n* = 507)	Girls mean (SD) (*n* = 476)
Age (years)	10.03 (1.41)	11.85 (2.55)	12.17 (2.54)
Height (cm)	145.15 (11.76)	155.90 (16.83)	153.7 (13.4)
Weight (kg)	41.36 (13.74)	51.77 (19.51)	48.98 (15.16)
BMI^a^ (score)	19.41 (6.44)	20.8 (7.03)	20.32 (4.22)
BFI^b^ (score)	13.85 (5.54)	10.62 (5.01)	16.19 (4.51)
Sport practice	*N* = 476 (48.4%)	*n* = 261 (54.8%)	*n* = 215 (42.3%)

^a^ BMI, body mass index.

^b^ BFI, body fat index.

Table 2 presents the incidence of spinal and foot postural deficiencies in entire cohort, as well as gender-specific comparisons. Within the entire group, forward head posture was the most prevalent spinal deficiency (641/983, 65.2%). According to gender, forward head posture was the most common deficiency in both boys (545/983, 63.6%) and girls (572/ 983, 62.9%), while ankle valgus represented the most common foot deficiency in boys (387/983, 45.2%) and in girls (388/983, 42.6%).

**Table 2:** The prevalence of spine and feet preadolescents postural deficiency for total group and according to gender in Bihor County, Romania, 2024.

Postural deficiency	The whole group (*n* = 983)	Boys (*n* = 507)	Girls (*n* = 476)	*p*
Kp^a^	510 (51.9%)	448 (52.3%)	368 (40.4%)	0.000
Sc^b^	257 (26.1%)	209 (24.4%)	306 (33.6%)	0.000
HFP^c^	641 (65.2%)	545 (63.6%)	572 (62.9%)	0.55
HL^d^	298 (30.3%)	169 (19.7%)	169 (21.5%)	0.34
AnVlg^e^	5,498 (0.5%)	387 (45.2%)	388 (42.6%)	0.28
FF^f^	459 (46.7%)	330 (38.5%)	359 (39.5%)	0.68

^a^ Kp, kyphosis.

^b^ SC, scoliosis.

^c^ HFP, head forward posture.

^d^ HL, hyperlordosis.

^e^ AnVlg, ankle valgus.

^f^ FF, flat feet.

In the preadolescent boys, the most prevalent postural deficiencies are head forward posture (545/983, 63.6%), followed by kyphosis (448/983, 52.3%), and ankle valgus (330/983, 38.5%). In the preadolescent girls, also the most prevalent postural deficiencies are head forward posture (572/983, 62.9%), followed by ankle valgus (388/983, 42.6%) and kyphosis (368/893, 40.4%).

The comparison analysis between preadolescent boys and girls showed that there is a significand difference in the prevalence of kyphosis in boys *n* = 448 (52.3%) comparing to girls *n* = 368 (40.4%) [*x^2^*(*1*) = 24.87, *p*˂0.001], and in the prevalence of scoliosis in girls *n* = 306 (33.6%) comparing to boys *n* = 257 (26.1%), [*x^2^*(*1*) = 18.24, *p*˂0.001]. There is no significant difference between groups regarding the head forward posture [*x^2^* (*2*) = 1.187, *p* = 0.55]. Hyperlordosis [*x^2^*(*1*) = 0.891, *p* = 0.34], calcaneus valgus [*x^2^*(*1*) = 0.113, *p* = 0.28] and flat feet [*x^2^*(*1*) = 0.165, *p* = 0.68].

Intra-rater reliability of all spine and feet postural deficiency (kyphosis, scoliosis, head forward posture, hyperlordosis, ankle valgus and flat feet) showed moderate results, with Cohen's kappa (k) values for inter-rater reliability of all spinal postural deficiencies ranged from 0.356 to 0.579, showing no statistical difference in measurements between different raters.

**Figure 2:**
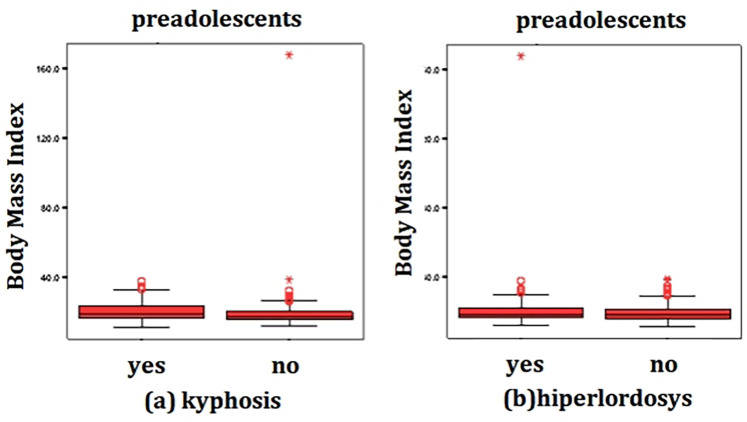
Correlation between BMI and the presence of kyphosis (**a**) and hyperlordosis (**b**) in preadolescents (*n* = 983), in Bihor County, Romania, 2024.

In the total cohort, point-biserial correlation analysis indicated a weak positive association between BMI and scoliosis (*r* = .123, *n* = 983, *p* < 0.001), calcaneus valgus (*r* = .163, *n* = 983, *p* < 0.001) and flatfoot (*r* = .054, *n* = 983, *p* = 0.007). This means that there is a weak inverse association between elevated BMI and the presence of scoliosis and calcaneus valgus. A weak negative correlation was found between BMI and kyphosis (*r* = −.072, *n* = 983, *p* < 0.001) while hyperlordosis showed an even weaker positive correlation (r = .020, *n* = 983, *p* = 0.009) meaning that a high BMI is poor associated with kyphosis and hyperlordosis (Figure 2). No significant correlation was observed between BMI and forward head posture (*r* = −.050, *n* = 983, *p* = 0.11).

**Figure 3:**
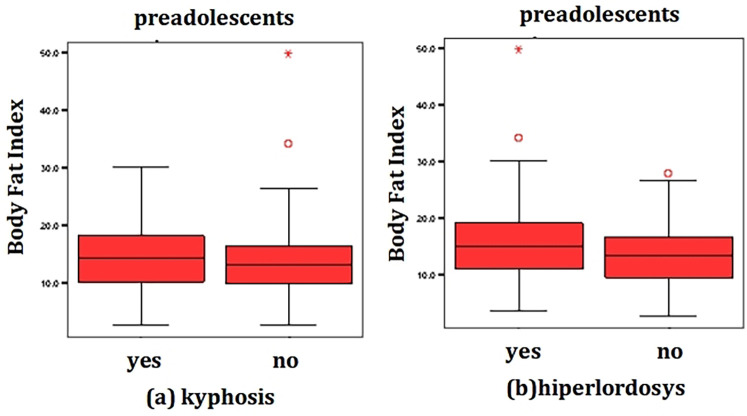
Correlation between BFI and the presence of kyphosis (**a**) and hyperlordosis (**b**) in preadolescents (*n* = 983), in Bihor County, Romania, 2024.

Point biserial correlation analysis of BFI demonstrated a weak positive correlation with ankle valgus (*r* = .073, *n* = 983, *p* < 0.001) and weak negative correlation with the presence of kyphosis (*r* = −.095, *n* = 983, *p* = 0.003) and hyperlordosis (*r* = −.162, *n* = 983, *p* < 0.001) (Figure 3). No significant correlations were identified between BFI and scoliosis (*r* = .014, *n* = 983, *p* = 0.66), forward head posture (*r* = −.023, *n* = 983, *p* = 0.46), or flatfoot (*r* = −.023, *n* = 983, *p* = 0.47). These results indicate that higher BFI showed a weak inverse association with ankle valgus and a weak positive association with kyphosis.

Analyses revealed a significant correlation between ankle valgus and scoliosis [*x^2^*(*1*) = 7.87, *p* = 0.005]. No significant associations were found coordination level [*x^2^*(*3*) = 3.69, *p* = 0.15], forward head posture [*x^2^*(*1*) = 1.20, *p* = 0.77], kyphosis [x^2^(*2*) = .306, *p* = 0.23], or hyperlordosis [*x^2^*(*1*) = 0.108, *p* = 0.15]. Flatfoot was significantly associated with both coordination [*x^2^*(*3*) = 22.96, *p* = 0.005] and scoliosis [*x^2^*(*1*) = 12.18, *p* < 0.001], but not with kyphosis [*x^2^*(*1*) = 2.70, *p* = 0.23], forward head posture [*x^2^*(*2*) = 6.38, *p* = 0.38], or hyperlordosis [*x^2^*(*1*) = 2.71, *p* = 0,27].

Table 3 presents the comparison of coordination and center of gravity (COG) displacement between boys and girls. Significant gender difference were observed for anterior displacement (COGA) [*t*(*981*)=−2.65, *p* = 0.005] and posterior displacement (COGP) [*t*(*981*) 753.24 = 2.309, *p* = 0.02]. No significant difference were found for rightward Matorin turns (RMT) [*t*(782)=−0.82, *p* = 0.056], leftward Matorin turns (LMT) [*t*(782)=−0.40, *p* = 0.13], rightward COG displacement (COGR) [*t*(*981*) = 0.89, *p* = 0.314], or leftward COG displacement (COGL) [*t*(*981*) = 0.60, *p* = 0.560].

**Table 3:** Comparison between boys (*n* = 507) and girls (*n* = 476) regarding the results of coordination and center of gravity displacement, in Bihor County, Romania, 2024.

Assessment tests	Boys mean (SD)	Girls mean (SD)	*p*
RMT^a^ (°)	314.01 (64.60)	314.25 (58.40)	0.056
LMT^b^ (°)	313.51 (63.21)	314.68 (58.53)	0.137
COGA^c^	46.80 (8.47)	47.85 (8.23)	**0**.**005**
COGP^d^	52.74 (8.57)	51.81 (8.39)	**0**.**021**
COGR^e^	51.09 (18.52)	50.51 (5.75)	0.314
COGL^f^	49.25 (6.04)	49.27 (5.92)	0.560

Bold values indicate statistically significant.

^a^ RMT, right matorin test.

^b^ LMT, left matorin test.

^c^ COGA, anterior displacement of center of gravity.

^d^ COGP, posterior displacement of center of gravity.

^e^ COGR, right displacement of center of gravity.

^f^ COGL, left displacement of center of gravity.

The logistic regression model was statistically significant for kyphosis [*χ*^2^(3) = 58.299, *p* < .0005], hyperlordosis [*χ*^2^(3) = 42.37, *p* < .0005], scoliosis [*χ*^2^(3) = 60.445, *p* < .0005], and calcaneus valgus [*χ*^2^(3) = 11.410, *p* < .0005].

For kyphosis, the Hosmer-Lemershow test indicated a good model fit [x^2^(8) = 14.86, *p* = .062]. The model explained of the variance 70.0% (Nagelkerke R2) and correctly classified 62.4% of cases with sensitivity of 65.8%, specificity of 58.8%, positive predictive value of 63.20% and negative predictive value of 61.5%. All three predictor—age, gender, and BMI—were statistically significant. Boys had 5.19 times lower odds of exhibiting kyphosis compared with girls (B = −.655, Wald = 24.76, *p* = .000; Exp(B) = .519, 95% CI [.401,.672]. Increasing age [B = −.125, Wald = 6.83, *p* = .009; Exp(B) = 1.134, 95% CI [1.032, 1.245]] and BMI [B = −.085, Wald = 26.71, *p* = .000. Exp(B) = .919, 95% CI [.889, .949]] were also associated with an increased likelihood of kyphosis.

For hyperlordosis, the Hosmer-Lemershow test also suggest a good fit [x^2^(8) = 10.67, *p* = .221]. The model explained 60.0% of the variance (Nagelkerke R2) and correctly classified 69.7% of cases, with sensitivity of 5.7%, specificity of 99.7%, positive predictive value of 98.47% and negative predictive value of 29.17%. Age and BMI were statistically significant predictors. Increasing age [B = .304, Wald = 32.44, *p* = .000; Exp(B) = 1.355, 95% CI [1.221, 1.504]] and BMI [B = −.057, Wald = 12.23, *p* = .000; Exp(B) = .944, 95% CI [.915,.975]] were associated with a higher likelihood of hyperlordosis.

For scoliosis, the Hosmer-Lemershow test indicated adequate model fit [x^2^(8) = 8.634, *p* = .374]. The model explained 87.3% of the variance (Nagelkerke R2) and correctly classified 73.9% of cases, with sensitivity of 8%, specificity of 99.3%, positive predictive value of 15.7% and negative predictive value of 26.05%. BMI (B = −.108, Wald = 26.60, *p* = .000. Exp(B) = 1.114, 95% CI [1.069, 1.161] and gender [B = .802, Wald = 17.88, *p* = .000; Exp(B) = 2.230, 95% CI [1.656, 3.004]] emerged as significant predictors. Boys had 2.23 times higher odds of scoliosis compared with girls, and higher BMI was associated with greater likelihood of scoliosis.

For calcaneus valgus the Hosmer-Lemershow test also showed good model fit [x^2^(8) = 11.148, *p* = .193]. The model explained 20% of the variance (Nagelkerke R2) and correctly classified 64.4% of cases. Sensitivity was 0%, while specificity was 100%. BMI [B = −.057, Wald = 9.661, *p* = .002. Exp(B) = 1.056, 95% CI [1.021, 1.089]], indicating that children with higher MBI demonstrated 1.05 times greater of presenting calcaneus valgus.

## Discussion

This study aimed to estimate the prevalence of spinal and foot postural deficiencies among preadolescents and to explore correlations between posture, body composition, coordination, and foot conditions in relation to spinal deviations.

The most prevalent spinal deficiency identified was forward head posture, observed across the entire cohort. Considering that the head constitutes approximately 8% of total body weight, proper alignment is critical for musculoskeletal health (29). Misalignment may lead to dysfunction in the head, neck, trunk, and upper limbs (29). Reported prevalence rates of forward head posture vary by age: Szczygieł et al. found that only 7.6% maintained correct head posture (29, 30), whereas Ruivo et al. documented a prevalence of 68% among adolescents aged 15–17 years (31). Such discrepancies likely reflect age-related differences in postural adaptation.

With respect to kyphotic posture, an overall prevalence of 51.9% was recorded, with boys (52.3%) more affected than girls (40.4%). This finding is consistent with previous studies reporting a higher prevalence of kyphotic posture among boys (32–35). However, other research has identified no significant gender differences (36), or has reported a higher prevalence of other spinal deformities among girls (37, 38).

Ankle valgus emerged as the most common lower limb postural deficiency, followed by flatfoot, with both conditions affecting boys and girls at similar rates. If uncorrected, ankle valgus may progress to secondary deformities such as hallux valgus, metatarsalgia, and osteoarthritis (39, 40), and it is often associated with flatfoot during growth phases (40, 41). Findings regarding flatfoot prevalence remain inconsistent: Xu et al. reported higher incidence in boys (42), whereas Sadeghi-Demneh observed no gender difference but documented a decline with age (43). Given these inconsistencies, the results suggest that the particularities related to sex between boys and girls during the early school years exert minimal influence on the development of postural and foot abnormalities. Supporting this, no major anthropometric or strength differences exist between prepubescent boys and girls (44).

A significant but weak correlation was observed between BMI, BFI, and the presence of kyphosis and hyperlordosis. This finding is consistent with prior research by Miranda et al. (45) and others, which identified associations between increased BMI and both thoracic kyphosis and lumbar lordosis (46–51). However, the correlations in the present study were weaker, possibly due to the younger age of participants compared to earlier studies that examined older adolescents.

Another important observation was the association between ankle valgus, flatfoot, and scoliosis. Prior research has also demonstrated relationship between calcaneal valgus angle and scoliosis (52), as well as between flatfoot and spinal deformities (53). These association are thought to result from kinetic chain disruptions caused by excessive foot pronation, which alters musculoskeletal alignment (54). Children with flatfoot frequently exhibit altered center of gravity shifts, potentially contributing to spinal asymmetries (55).

Flatfoot is further recognised as negatively affecting daily activities and quality of life in children (56). Sung et al. affecting that individuals with symptomatic flatfoot develop compensatory movement patterns that impair postural stability (57). Although balance was not directly assessed in the present study, a significant link between flatfoot and body coordination was observed. Similar findings were reported by Takabayashi et al., who noted impaired coordination during running in children with flatfoot (58).

### Limitations

This study has several limitations. First, postural deviations were assessed through visual inspection using a plumb line. Although this method is widely applied in clinical and field settings owing to its simplicity and low cost, it is inherently subjective and may be influenced by the examiner's experience and judgment, thereby affecting the reliability and accuracy of the findings. The absence of precise quantitative measurements also limits the detection of subtle deviations and reduces comparability with studies of greater accuracy. Moreover, the correlation analysis was based on the number of children presenting deficiencies rather than the degree of postural deviation relative to BMI, which may have influenced the strength of the associations. To address this limitation, future research should consider incorporating more objective assessment tools, such as computerized postural analysis systems that capture images and allow for angular measurements of postural deviations, thereby ensuring greater reliability and reproducibility.

Nonetheless, visual observation remains a rapid, cost-effective, and non-invasive method suitable for large-scale screening. A further limitation is the exclusion of potentially relevant factors such as genetics, hormonal status, and nutrition, which may also influence posture. Finally, children's emotional discomfort during assessment—being outside their familiar environment and during assessment—being outside their familiar environment and evaluated in partial undress—may have affected posture evaluation.

## Conclusions

This study identified forward head posture as the most prevalent postural deficiency among preadolescents, followed by kyphotic posture and ankle valgus. Body composition was significantly associated with the presence of kyphosis, hyperlordosis, ankle valgus, and flatfoot, with flatfoot also linked to scoliosis and impaired general coordination. These findings underscore the importance of early detection of postural deficiencies and the implementation of targeted preventive and corrective programmes for school-aged children. Such initiatives are essential to reduce the risk of subsequent musculoskeletal disorders in the adult population and to promote healthier physical development trajectories into adulthood.

## Data Availability

The raw/processed data required to reproduce the above findings cannot be shared at this time as the data also forms part of an ongoing study. Requests to access the datasets should be directed to dciobanu@uoradea.ro.
